# The Herbal Pair *Polygonum hydropiper* *L‐Coptis chinensis Franch* Attenuate DSS‐Induced Ulcerative Colitis by Modulating Metabolism and Intestinal Flora

**DOI:** 10.1002/fsn3.70584

**Published:** 2025-07-08

**Authors:** Feifei Zhu, Chenhui Ren, Keli Zhou, Xueqing Huang, Mengyu Mei, Haiyan Niu, Shouzhong Ren

**Affiliations:** ^1^ Department of Pathology, The First Affiliated Hospital and School of Basic Medicine and Life Science Hainan Medical University Haikou China; ^2^ Engineering Research Center of Tropical Medicine Innovation and Transformation of Ministry of Education, International Joint Research Center of Human‐Machine Intelligent Collaborative for Tumor Precision Diagnosis and Treatment of Hainan Province, Hainan Provincial Key Laboratory of Research and Development on Tropical Herbs, School of Pharmacy Hainan Medical University Haikou China; ^3^ School of Life Sciences Hainan University Haikou China

**Keywords:** 16S rDNA, intestinal flora, metabolomics, *polygonum hydropoper L‐Coptis chinensis Franch*, ulcerative colitis

## Abstract

Ulcerative colitis (UC) is a chronic inflammatory bowel disease in which dysbiosis of the intestinal flora plays a critical role in its pathogenesis. *Polygonum hydropiper L‐Coptis chinensis Franch* (PH‐CC) known for its anti‐inflammatory properties and ability to regulate gut microbiota, has been demonstrated to alleviate UC. The aim of this study was to investigate the effects of PH‐CC on dysbiosis of intestinal flora and metabolic disorders in dextran sodium sulfate (DSS)‐induced UC mice. A UC mouse model was induced using a 3% DSS solution, and histopathological changes in the colon were assessed through hematoxylin and eosin staining. The composition and diversity of the intestinal flora were analyzed using 16S rDNA technology, whereas metabolomics was employed to identify potential differential metabolites and metabolic pathways through multivariate statistical analysis. Pearson correlation analysis was performed between metabolites, inflammatory factors, the NLRP3/Caspase‐1 pathway, and differential flora. The results revealed that treatment with PH‐CC improved the morphological structure of colon tissue and reduced damage in UC mice, while the model group exhibited significant damage to the crypt structure, exfoliation of the colonic mucosal epithelium, loss of glands, and infiltration of inflammatory cells. Analysis of 16S rDNA sequencing data indicated that PH‐CC regulated the DSS‐induced changes in gut microbiota, significantly decreasing the abundance of *norank_f_norank_o_Rhodospirillales, norank_f_UCG‐010, Corynebacterium, and Eubacterium_nodatum_group.* The differential microbiota exhibited a strong correlation with the NLRP3/Caspase‐1 pathway and downstream inflammatory factors. Additionally, 27 differential metabolites identified in fecal samples were primarily associated with phenylalanine metabolism and bile secretion, showing a high correlation with the differential microbiota. In conclusion, PH‐CC regulates dysbiosis in intestinal flora and metabolic disorders in UC mice by reducing the abundance of specific bacterial groups, thereby alleviating inflammatory damage to the colonic mucosa and improving the overall condition of UC.

## Graphical abstract

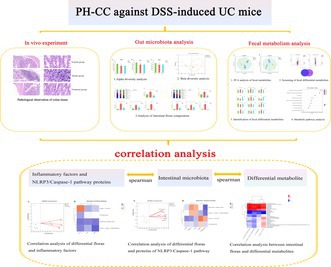

## 1 Introduction

Ulcerative colitis (UC) is a chronic inflammatory bowel disease characterized by mucosal inflammation and damage to the colonic wall, often leading to symptoms such as abdominal pain, diarrhea, and hematochezia (Voelker 2024; Le Berre et al. 2023). The prevalence of UC is increasing across various regions of Asia, particularly in China, where it has emerged as a common digestive disorder (Liu et al. 2022; Buie et al. 2023). As of 2023, the prevalence of UC was estimated to be 5 million cases around the world, and the incidence is increasing worldwide (Le Berre et al. 2023). In China, the number of UC cases is projected to exceed 1 million by 2025, underscoring the growing burden of this condition. Although the precise etiology of UC remains uncertain, it is believed to be influenced by environmental factors, microbiota imbalance, and immune system dysfunction (Bruland et al. 2021; Poppe et al. 2024; Zhang and Zhao 2022). Current treatment options for UC are limited, primarily focusing on symptom relief and inflammation reduction (Danese et al. 2025). While medications can offer some degree of relief, they may also lead to serious side effects, including disease recurrence, infections, and even tumor development. Traditional Chinese medicine, known for its treatment approach based on syndrome differentiation and its favorable safety profile, is increasingly being explored as a potential therapeutic option for UC (Xu et al. 2025; Liang et al. 2024; Zheng et al. 2025).

The herbs *Polygonum hydropiper* L and *Coptis chinensis Franch* exhibit various biological activities, including anti‐inflammatory, antioxidant, and digestive protective properties, which render them effective in treating inflammatory bowel diseases (Chen et al. 2020; Xie et al. 2022; Yang, Vong, et al. 2021; Ai et al. 2021). Herbal compatibility is a fundamental principle and essence of traditional Chinese medicine prescriptions, facilitating synergistic effects while minimizing toxicities and side effects (Gao et al. 2022; Chen et al. 2025). Our experimental study demonstrated that the combination of PH‐CC can inhibit the abnormal activation of the NLRP3/Caspase‐1 pathway, reduce the release of IL‐18 and IL‐1β, alleviate damage to the colonic mucosa, and ultimately mitigate UC in mice (this aspect of the content has already been published). However, the pharmacological effects of traditional Chinese medicine are complex, involving multi‐channel, multi‐link, multi‐target, and integrated regulation, which complicates the elucidation of the mechanisms underlying UC treatment in a singular manner. Furthermore, the persistence and progression of UC arise from a combination of intricate etiological factors rather than a single cause (Xu et al. 2023). Recent studies have confirmed a strong association between dysbiosis of intestinal flora and the development of UC (Singh et al. 2025; Shi et al. 2025; Świrkosz et al. 2023).

Disruption of flora can trigger a cascade of immune responses in the host's intestinal mucosa, potentially leading to inflammatory bowel disease (Qiu et al. 2022; Yan and Wu 2023). Changes in the composition and function of the intestinal flora can impact the host's metabolic responses, while the host's metabolic state can reciprocally influence the composition and function of the intestinal flora. Currently, there are no reports regarding whether the effects of PH‐CC in treating UC are linked to alterations in intestinal flora and metabolites. This study aims to examine how PH‐CC affects UC by analyzing metabolomics and intestinal flora, thereby investigating the potential mechanism for improving UC from various perspectives.

## 2 Material and Methods

### 2.1 Animals and Reagents

Balb/c mice (18–22 g) were obtained from Changsha Tianqin Biotechnology Co. Ltd., China (SCXK (xiang) 2022‐0011) and were housed in a well‐ventilated SPF‐grade animal facility maintained at a temperature of 22°C ± 2°C, with humidity levels ranging from 40% to 60%, and a 12‐h light–dark cycle. Following a 1‐week acclimatization period, the mice were randomly divided into three groups: the normal group, the model group, and the PH‐CC group (114 mg/kg). The procedures for the animal experiments were conducted in strict compliance with the guidelines for the treatment and utilization of laboratory animals and received approval from the Ethics Committee of Hainan Medical University (HYLL‐2024‐643).

Dextran sulfate sodium (DSS) was acquired from MP Biomedicals (no. 160110 Aurora, OH, USA). The DNA Gel Recovery and Purification Kit was sourced from Shanghai Major Bio‐Pharm Technology Co. Ltd. (no. C01‐10000, Shanghai, China). The NEXTFLEX Rapid DNA‐Seq Kit was purchased from Bio Scientific (no. NOVA‐5144, USA). OMG‐Soil was obtained from Omega Bio‐Tek (no. M9636‐02, Georgia, USA).

### 2.2 Source and Preparation of PH‐CC Extracts

*Polygonum hydropiper* L was gathered in Wuzhishan City, Hainan, China, and identified by Prof. Niankai Zeng of Hainan Medical University. The voucher specimen (no. 20191016) was subsequently stored in the Laboratory of Traditional Chinese Medicine at the School of Pharmacy, Hainan Medical University. Coptis chinensis Franch (no. 220201) was supplied by Shijiazhuang Chengxin Traditional Chinese Medicine Co. Ltd. in Hebei, China, and was also identified by Prof. Niankai Zeng of Hainan Medical University.

The PH‐CC mixture, prepared in a 2:1 ratio, was soaked overnight in 60% ethanol and then subjected to heating reflux extraction twice. The filtrate was combined after suction filtration, concentrated under reduced pressure, purified by column chromatography, and concentrated again under reduced pressure before being dried into a powder for storage.

### 2.3 Modeling and Treatment

All groups, with the exception of the normal group, were administered a 3% DSS solution to drink for a duration of 10 days. The PH‐CC group, however, received continuous intragastric administration for 9 days, commencing on the second day. On the 11th day, the mice were anesthetized using a 1% pentobarbital sodium solution to facilitate the collection of feces and colon tissues.

### 2.4 Antioxidant Activity

#### 2.4.1 DPPH Determination

A total of 2 mL of various concentrations of the PH‐CC solution was taken, followed by the addition of 2 mL of a 0.06 mg/mL DPPH solution. The mixture was then incubated in the dark for 2.5 h. Anhydrous ethanol served as a blank control in place of DPPH. The absorbance value *A*1 was measured at 517 nm, while anhydrous ethanol was also used to adjust the absorbance value *A*0 at 517 nm to zero. Additionally, 2,6‐Di‐tert‐butyl‐4‐methylphenol (BHT) served as a positive control. The clearance rate was calculated using the formula:

$$\text{Clearance rate}=\left(A0-A1\right)/A0\times 100\%$$

where *A*1 is the absorbance value of sample group and *A*0 is the absorbance value of the control group.

#### 2.4.2 Determination of Hydroxyl Radical Scavenging Rate

Different concentrations of PH‐CC solutions (1 mL) were added to a test tube, followed by the sequential addition of 0.3 mL of 9 mmol/L FeSO4 solution and 0.3 mL of 9 mmol/L salicylic acid‐ethanol solution. The solution was then brought to a total volume of 6 mL with distilled water. After thorough mixing, 0.3 mL of an 8.8 mmol/L H_2_O_2_ solution was added to initiate the reaction, which was allowed to proceed at 37°C for 10 min. For the blank control, distilled water was used instead of the H_2_O_2_ solution. The absorbance value *A*1 was measured at 527 nm, and distilled water was also used to measure the absorbance value *A*0, which was adjusted to zero. Glutathione (GSH) served as the positive control. The clearance rate was calculated using the formula:

$$\text{Clearance rate}=\left(A0-A1\right)/A0\times 100\%$$

*A*1 is the absorbance value of the sample group and *A*0 is the absorbance value of the control group.

#### 2.4.3 Reduction Force Determination

A volume of 1 mL from various concentrations of the PH‐CC solution was added separately to 2.5 mL of 1% K3[Fe (CN)6] solution and 2.5 mL of a 0.2 mol/L phosphate buffer solution (pH 6.8). The samples were incubated at 50°C for 20 min, after which the reaction was halted by placing the samples in a refrigerator at 4°C for 5 min. Subsequently, 2.5 mL of 10% C_2_HCl_3_O_2_ solution was added to each sample, which was then centrifuged at 3000 rpm for 10 min. From the resulting supernatant, 2.5 mL was extracted, to which 2.5 mL of distilled water and 0.1 mL of FeCl_3_ solution were added. The absorbance was measured at 700 nm, with glutathione (GSH) serving as the positive control.

### 2.5 Pathological Observation of Colon Tissue

Colon tissue samples were collected from each group of mice and initially fixed with 4% paraformaldehyde to preserve tissue integrity throughout the subsequent procedures. The samples underwent a series of processing steps, including dehydration, embedding, sectioning, and staining. Finally, the prepared sections were examined and analyzed under a microscope.

### 2.6 16S rDNA Sequencing Analysis and Bioinformatics Analysis

DNA was extracted from fecal samples of mice in each group using standardized methods. Universal primers were either selected or designed for PCR amplification based on the characteristics of the samples. The resulting PCR products were purified, evenly distributed, and utilized to construct paired‐end (PE) libraries. The qualified library was then sequenced and analyzed using Illumina technology.

The raw data underwent processing on the Illumina sequencing platform, which included splicing, quality control, and filtering to yield optimized sequences. These optimized sequences were subjected to operational taxonomic unit (OTU) clustering, grouping sequences with 97% similarity, while chimeras were removed to create an OTU abundance table. Subsequently, analyses of alpha diversity, beta diversity, species community composition, and species differences within the intestinal flora of the mice were conducted based on the OTU abundance table.

### 2.7 Metabolomics Analysis Conditions and Sample Processing

A total of 25 mg of feces was collected from each group of mice and mixed with 300 μL of an extraction solution (methanol: water = 4:1). The samples were ground at −10°C for 6 min and subjected to ultrasonic extraction at low temperature for 30 min. Following this, the mixed solution was centrifuged at 4°C at 13,000 rpm for 15 min, and the supernatant was collected for analysis. Additionally, 20 μL of samples from each group were combined to create a quality control (QC) sample, which was included after every 3–5 test samples to assess system stability.

The chromatographic columns utilized were ACQUITY UPLC HSS T3 (100 mm × 2.1 mm id, 1.8 μm). The mobile phase A consisted of 5% acetonitrile and 95% water (containing 0.1% formic acid), while mobile phase B comprised 5% water, 47.5% acetonitrile, and 47.5% isopropanol (also with 0.1% formic acid). The injection volume was set to 2 μL, the column temperature was maintained at 40°C, and the flow rate was established at 0.4 mL/min. The gradient elution profile was as follows: at 0 min, 100% A; at 3 min, 80% A; at 4.5 min, 65% A; and from 5 to 8 min, a transition from 0% to 100% A.

The scan type ranged from 70 to 1050 m/z, with a sheath gas flow rate of 50 arb, an auxiliary gas flow rate of 13 arb, a heater temperature set at 425°C, and a capillary temperature of 325°C. The positive spray voltage was maintained at 3500 V, while the negative spray voltage was set at −3500 V. The S‐Lens RF Level was adjusted to 50 V, and normalized collision energy levels were set at 20, 40, and 60 eV.

### 2.8 Metabolomics Data Processing and Analysis

The raw data, including samples and quality control samples, were imported into Progenesis QI software for preprocessing to generate a data matrix. Subsequently, principal component analysis (PCA), partial least squares discriminant analysis (PLS‐DA), and differential metabolite analysis were performed. Differential metabolites were selected based on the following criteria: VIP > 1, FC > 1.2 or FC < 0.83. The identified differential metabolites were then imported into Scripty 1.0.0 software for metabolic pathway enrichment analysis, utilizing the HMDB database.

### 2.9 Statistical Analysis

All data are presented as the mean ± standard deviation (SD). Statistical analyses were conducted using the SPSS 23.0 software package. Comparisons among multiple groups were performed using one‐way analysis of variance (ANOVA), with *p* < 0.05 considered statistically significant.

## 3 Results

### 3.1 PH‐CC Has Good Antioxidant Activity

#### 3.1.1 DPPH Free Radical Scavenging Ability

**FIGURE 1:**
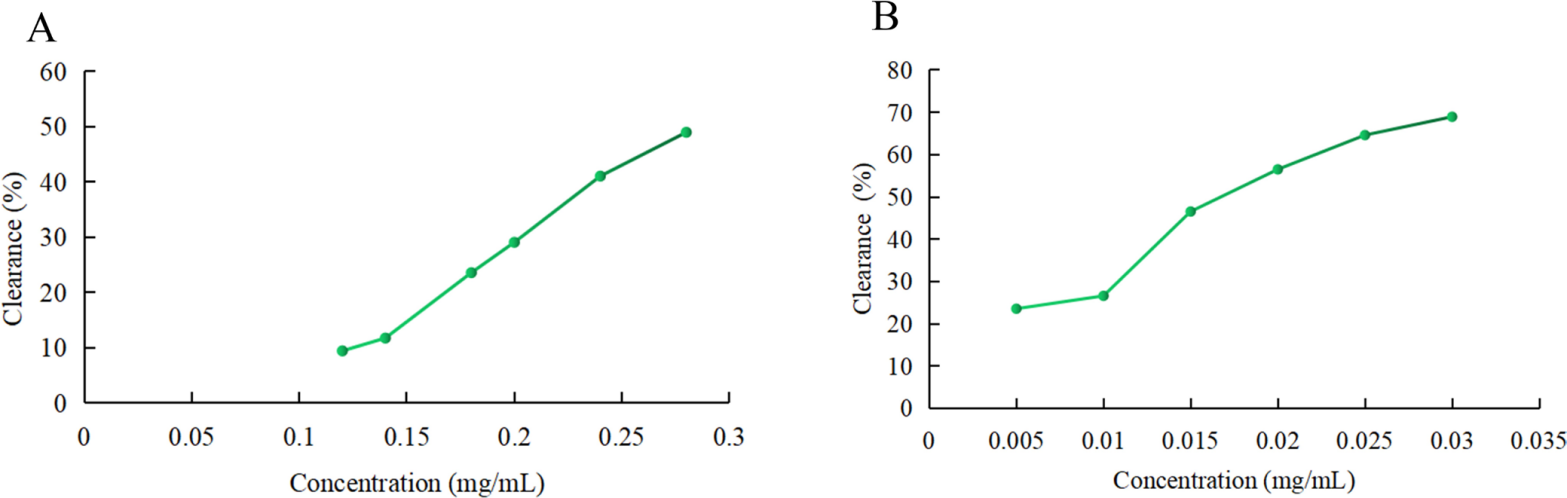
Effects of PH‐CC and BHT on DPPH radical scavenging rate: (A) PH‐CC and (B) BHT.

The scavenging effect of PH‐CC on DPPH free radicals is illustrated in Figure 1. Within the concentration range utilized in the experiment, the scavenging ability of PH‐CC on DPPH free radicals increases as the concentration increases. The IC50 value is 0.297 mg/mL; however, the scavenging rate is lower than that of BHT, which has an IC50 of 0.019 mg/mL.

#### 3.1.2 Hydroxyl Radical Scavenging Capacity

**FIGURE 2:**
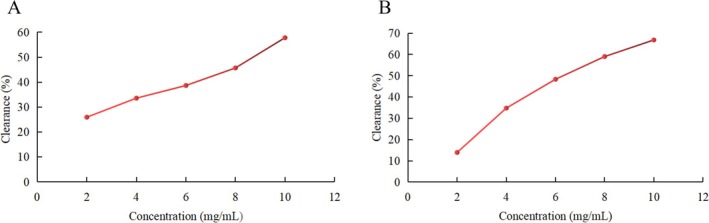
The effect of PH‐CC and GSH on the clearance rate of hydroxyl radical. (A) PH‐CC. (B) GSH.

The scavenging effect of PH‐CC on hydroxyl radicals is depicted in Figure 2 Within the concentration range of 2–10 mg/mL, the scavenging ability of PH‐CC on hydroxyl radicals also increases with concentration. The IC50 of PH‐CC is 8.552 mg/mL, while the IC50 of GSH is 6.840 mg/mL.

#### 3.1.3 Reduction Ability

**FIGURE 3:**
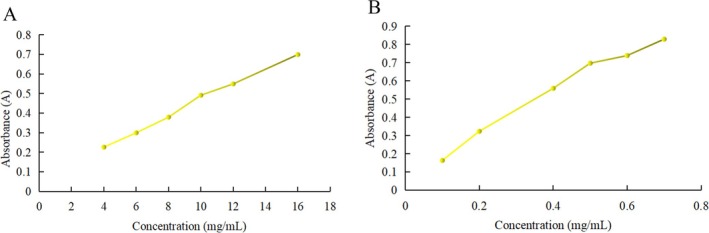
The reduction ability of PH‐CC and GSH. (A) PH‐CC and (B) GSH.

The reduction ability of PH‐CC is presented in Figure 3. The reduction capacity of a substance is closely related to its potential antioxidant activity. The reduction ability of PH‐CC increases with concentration, demonstrating a clear dose–effect relationship; however, it remains lower than that of glutathione at the same concentration.

### 3.2 PH‐CC Intervention Alleviates DSS‐Induced Colonic Mucosal Damage

**FIGURE 4:**
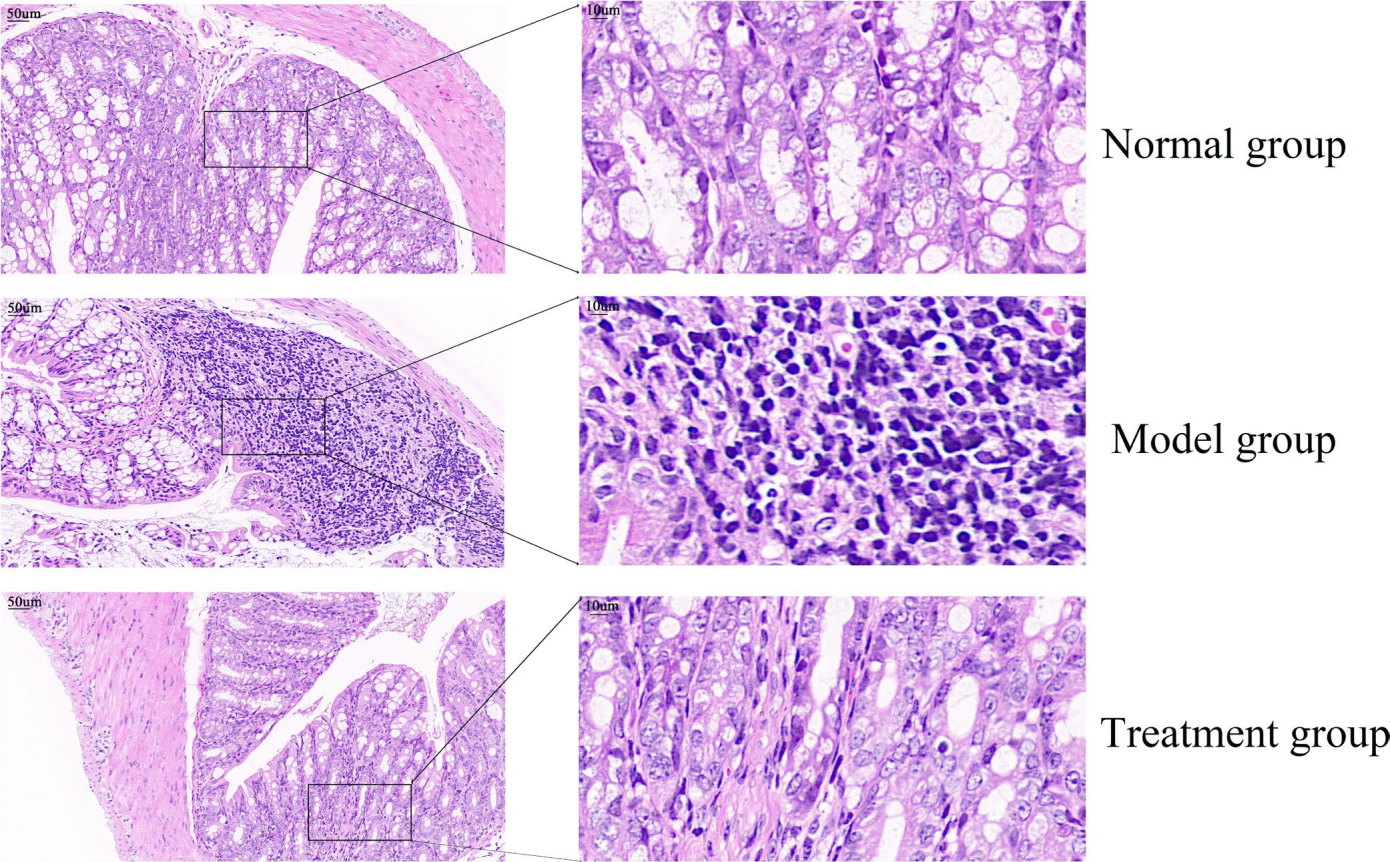
Pathological observation of colon tissue.

In the normal group, mice exhibited intact colonic mucosal tissue, well‐arranged glands, and no inflammatory cell infiltration. Conversely, the model group displayed significant destruction of the crypt structure, characterized by broken and detached colonic mucosal epithelium, absent glands, and a high level of inflammatory cell infiltration. Following treatment, the PH‐CC group exhibited less severe pathological injury compared to the model group (Figure 4).

### 3.3 PH‐CC Altered the Richness and Composition of the Gut Microbiota Community

**FIGURE 5:**
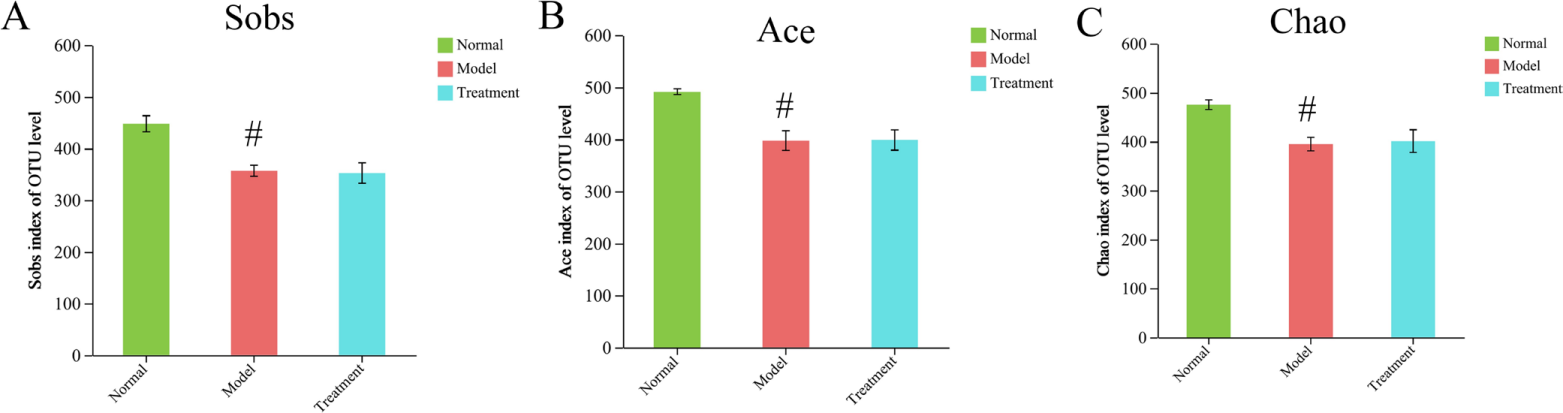
Analysis of Sobs (A), Ace (B), and Chao (C). ^*#*^ *p* < 0.05 versus normal group.

**FIGURE 6:**
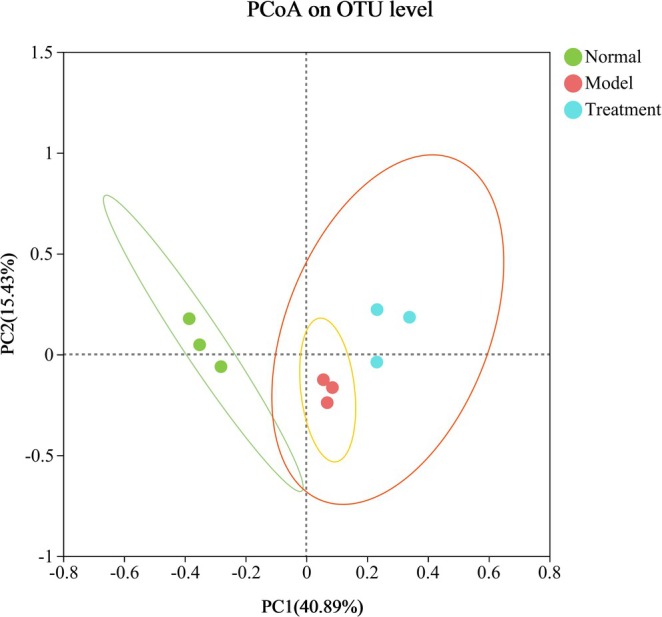
Diversity analysis of intestinal flora in mice.

Alpha diversity serves as a comprehensive measure of the richness and diversity of microbial species. Commonly employed evaluation indices include Shannon, Simpson, Chao, Ace, and Sobs. The species diversity and richness among groups are further examined through inter‐group difference tests. In comparison to the normal group, DSS‐induced UC mice exhibited a significant reduction in intestinal flora abundance. Moreover, no significant differences in alpha diversity were observed between the PH‐CC group and the model group (Figure 5). Consequently, beta diversity analysis suggests that PH‐CC treatment for UC is not significantly associated with the regulation of intestinal flora. The beta diversity in UC mice was analyzed using PCoA with the Bray–Curtis distance algorithm. The sample points between groups exhibited a high degree of separation, while sample points within the group were closely clustered, indicating a significant difference in overall flora composition among the three groups of mice (Figure 6).

**FIGURE 7:**
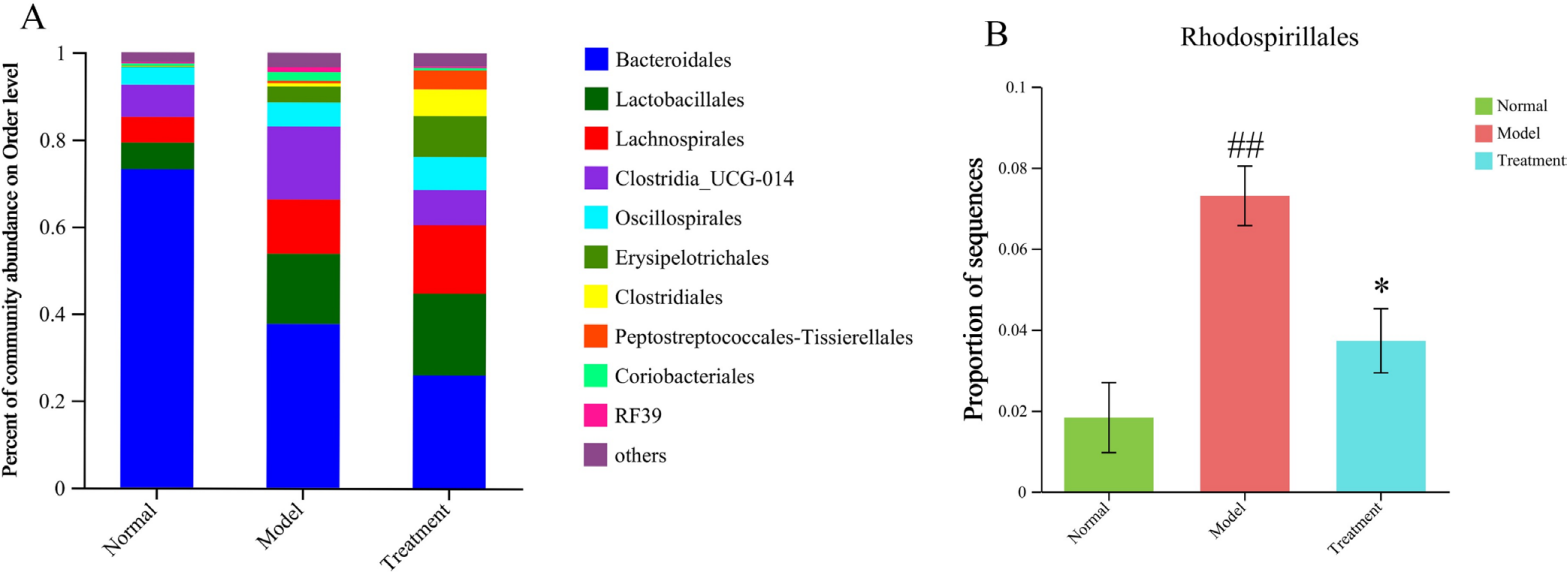
Order level (A) and species difference (B) analysis of intestinal flora in mice. ^##^ *p* < 0.01 versus normal group; *p < 0.05 versus model group.

**FIGURE 8:**
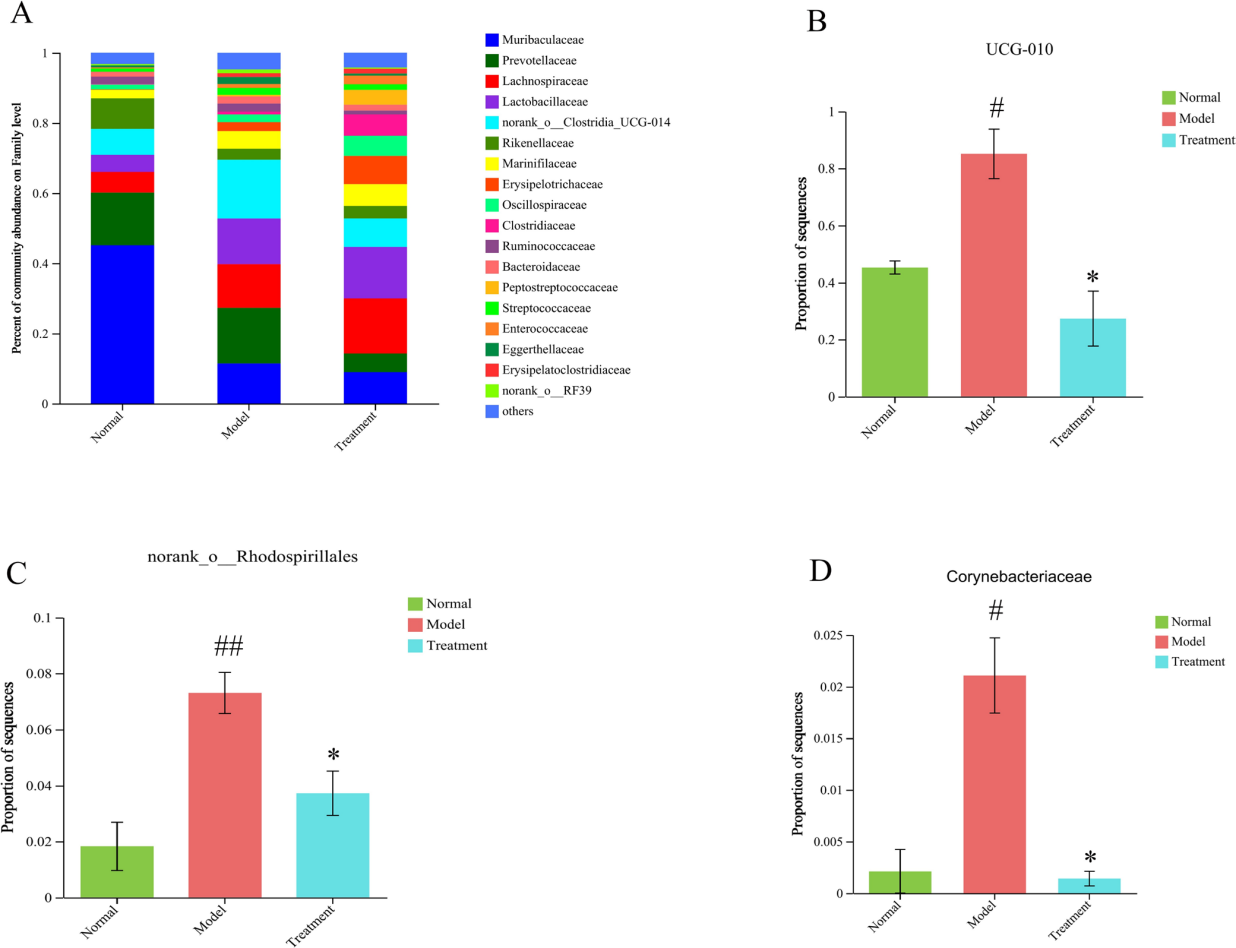
Analysis of intestinal floras at family level. (A) Analysis of main intestinal floras abundance proportion at family level. (B–D) Analysis of species differences at family level. ^#^ *p* < 0.05, ^##^ *p* < 0.01 versus normal group; *p < 0.05 versus model group.

**FIGURE 9:**
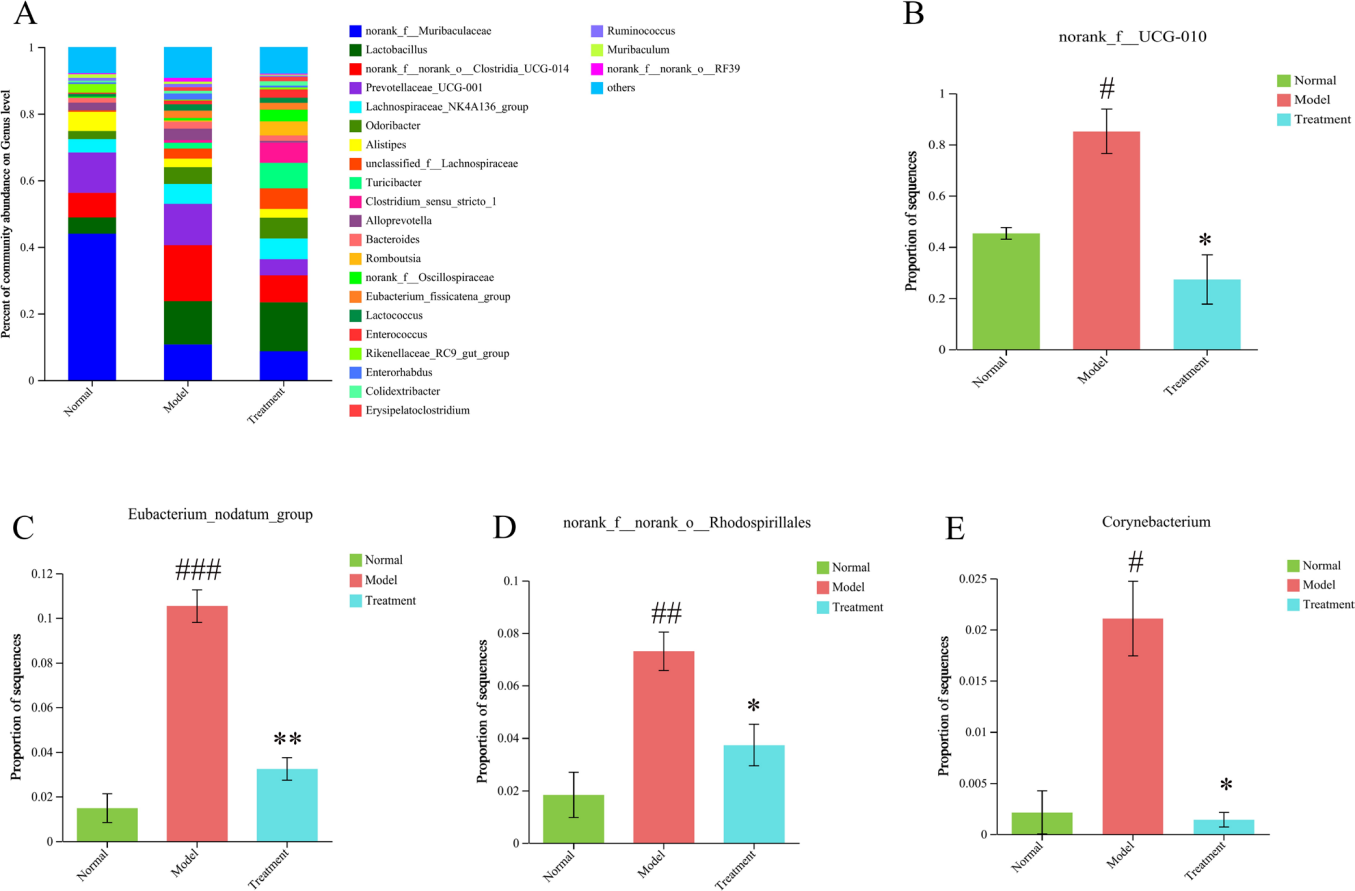
Analysis of intestinal floras at genus level. (A) Analysis of main intestinal floras abundance proportion at genus level. (B–E) Analysis of species differences at genus level. ^*#*^ *p* < 0.05, ^*##*^ *p* < 0.01, ^*###*^ *p* < 0.001: Normal versus model; *p < 0.05, **p < 0.01: Treatment groups versus model.

To investigate the impact of PH‐CC treatment on the composition of intestinal flora in UC, we analyzed the relative abundance of species at the order, family, and genus levels. Among the 10 orders examined, the abundance of intestinal flora exceeded 0.01. Compared to the normal group, DSS‐induced UC mice displayed a reduction in the relative abundance of *Rhodospirillales*. Notably, intervention with PH‐CC led to a significant reversal of the relative abundance of *Rhodospirillales* (Figure 7). In comparison to the normal group, the model group showed an increase in the relative abundance of *norank_o_Rhodospirillales, ruminococcaceae_UCG‐010*, and corynebacteriaceae at the family level. However, the PH‐CC intervention led to a significant decrease in the relative abundance of *ruminococcaceae_UCG‐010, norank_o_Rhodospirillales*, and corynebacteriaceae when compared to the model group (Figure 8). At the genus level, compared to the normal group, DSS‐induced UC mice showed an increased relative abundance of *norank_f_norank_o_Rhodospirillales, eubacterium_nodatum_group*, *corynebacterium*, and *norank_f_UCG‐010*. The intervention with PH‐CC resulted in a significant decrease in the relative abundance of these bacterial taxa (Figure 9). These findings suggest that PH‐CC may exert a therapeutic effect in mice with UC by modulating these microbial communities.

### 3.4 Correlation Analysis of Flora With Inflammatory Factors and NLRP3/Caspase‐1 Pathway

**FIGURE 10:**
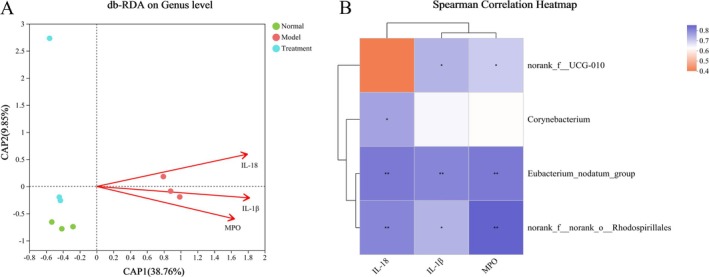
Correlation analysis of differential floras and inflammatory factors: (A) db‐RDA analysis and (B) heatmap analysis.

**FIGURE 11:**
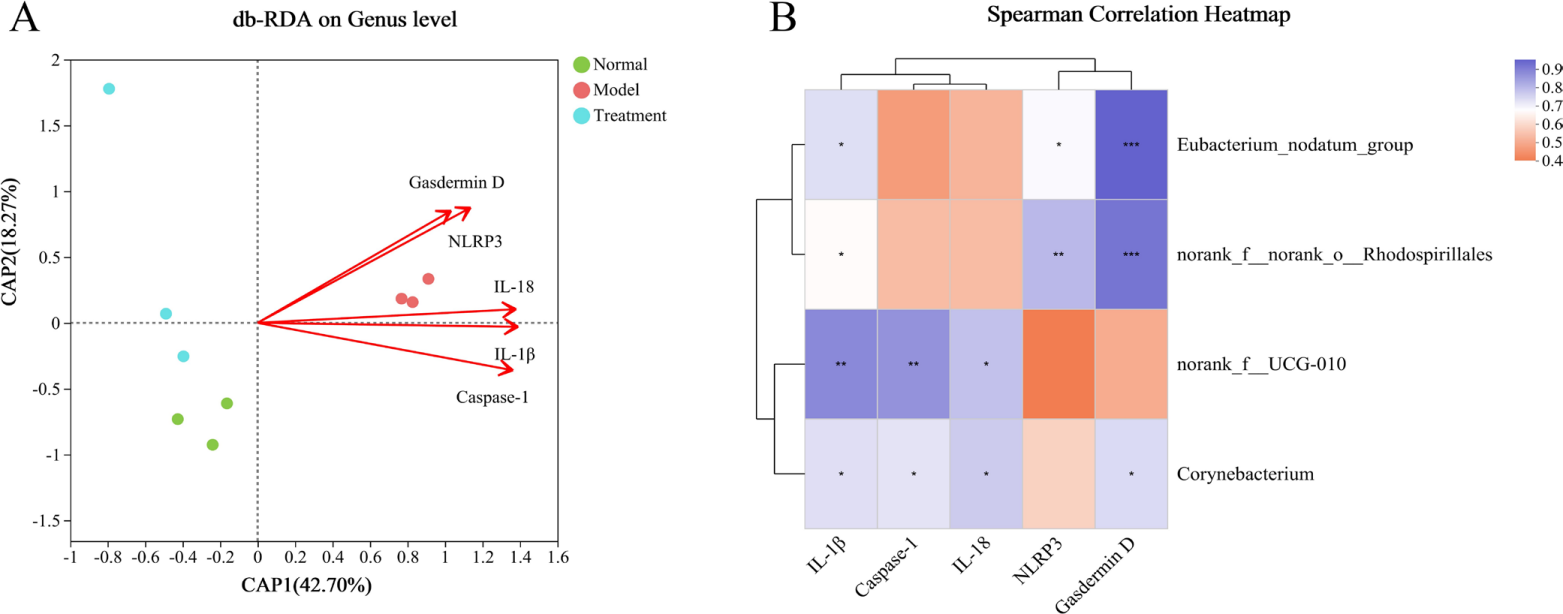
Correlation analysis of differential floras and proteins of NLRP3/Caspase‐1 pathway. (A) Db‐RDA analysis. (B) Heatmap analysis. *Note*: *p < 0.05, **p < 0.01, ***p < 0.001.

Differential floras at the genus level were analyzed along with environmental factors (IL‐18, IL‐1β, and MPO) using db‐RDA correlation analysis to identify key floras associated with variations in these environmental factors. Genera such as *eubacterium_nodatum_group, norank_f_UCG‐010, corynebacterium*, and *norank_f_norank_o_Rhodospirillales* exhibited a positive correlation with the levels of IL‐18, IL‐1β, and MPO. To provide a more visual representation of the correlation between environmental factors and differential floras, correlation heatmap plots were presented (Figure 10). Additionally, correlation analysis between differential floras at the genus level and environmental factors (NLRP3, Caspase‐1, Gasdermin D, IL‐18, and IL‐1β) was conducted using db‐RDA. The results indicated a positive correlation among *corynebacterium, norank_f_norank_o_Rhodospirillales, norank_f_UCG‐010*, and *eubacterium_nodatum_group* with the levels of NLRP3, Caspase‐1, Gasdermin D, IL‐18, and IL‐1β (Figure 11). This evidence suggests that PH‐CC protects colonic mucosa against DSS injury by downregulating the expression of the NLRP3/Caspase‐1 pathway and downstream key factors through the modulation of gut microbiota.

### 3.5 PH‐CC Modulates Mice Fecal Metabolism

**FIGURE 12:**
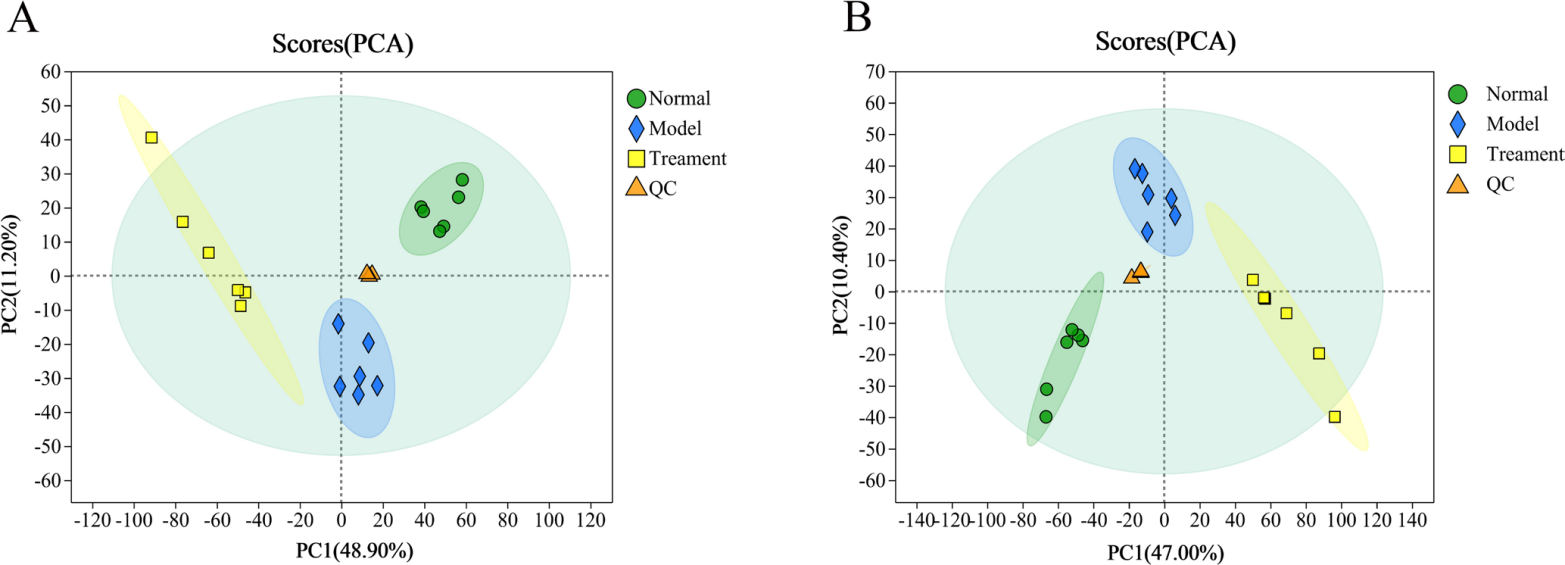
PCA analysis of fecal samples (A, B).

**FIGURE 13:**
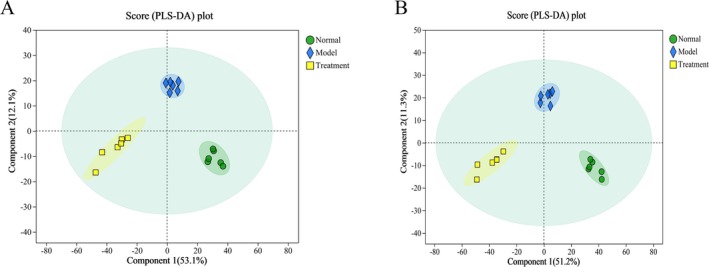
PLS‐DA analysis of fecal samples: (A) Positive ion mode; (B) negative ion mode.

Fecal samples were subjected to PCA and PLS‐DA analyses to investigate the impact of PH‐CC on fecal metabolism in UC mice. The PCA analysis revealed distinct separations between groups, with samples clustering within their respective categories, indicating notable differences in metabolite profiles among the three groups. In contrast, limited variation was observed within each group (Figure 12). Similarly, the PLS‐DA analysis demonstrated clear separations of samples, which also clustered within their respective groups (Figure 13). These results suggest that PH‐CC can effectively regulate the metabolism of UC mice in vivo. Furthermore, both *Q* ^2^ and *R* ^2^ values in the positive and negative ion modes exceeded 0.9, indicating a strong data fitting effect and predictive capability of the model.

**FIGURE 14:**
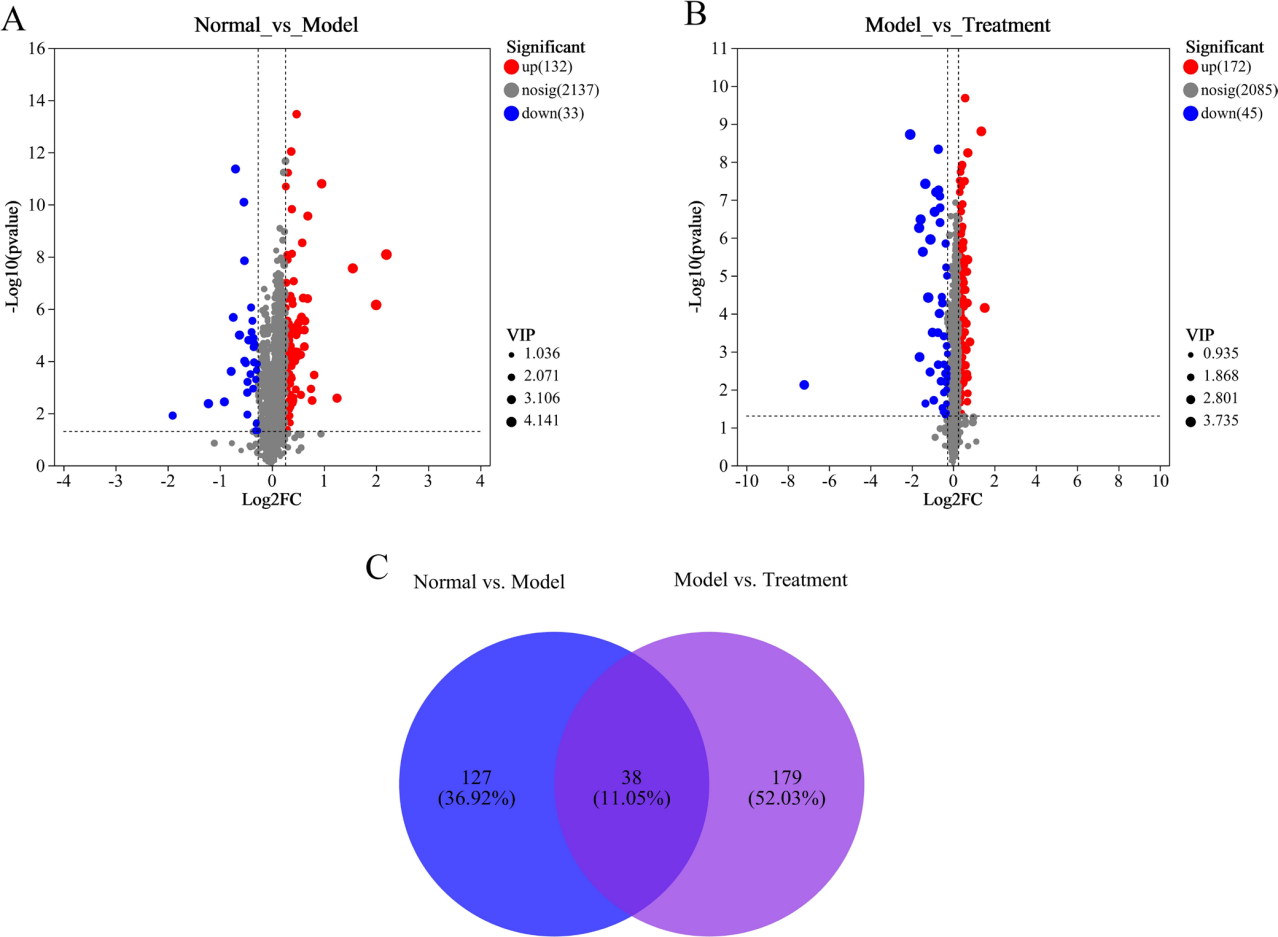
Differential metabolites analysis of mice fecal samples. (A) Normal versus model. (B) Model versus treatment. (C) Venn diagram of diferential metabolites in fecal samples.

**FIGURE 15:**
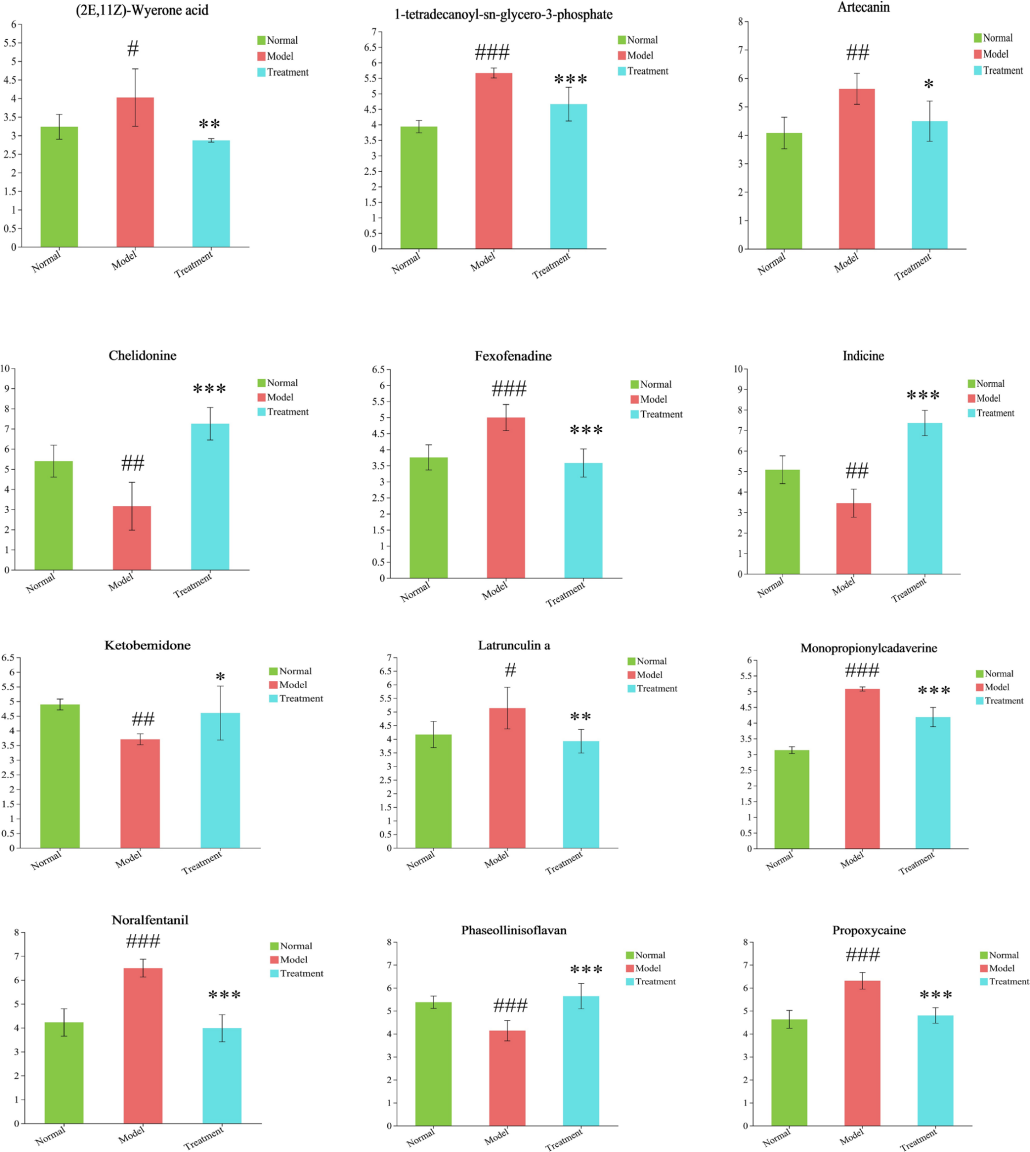
Differential metabolites contents of mice fecal samples. ^#^ *p* < 0.05, ^##^ *p* < 0.01, ^*###*^ *p* < 0.001 versus normal group; *p < 0.05, **p < 0.01, ***p < 0.001 versus model group.

The screening and identification of fecal differential metabolites were performed, identifying metabolites in each group based on the criteria of VIP > 1, *p* < 0.05, FC > 1.2, or FC < 0.83. Volcano plots visually represented the variations in metabolite levels between groups, highlighting statistically significant differences. A total of 165 differential metabolites were identified between the normal and model groups, with 132 upregulated and 33 downregulated in the model group (Figure 14A). Similarly, 217 differential metabolites were identified between the model and PH‐CC groups, with 172 upregulated and 45 downregulated in the PH‐CC group (Figure 14B). Furthermore, Venny analysis revealed 38 common differential metabolites across all three groups (Figure 14C). In total, 38 differential metabolites were identified and subsequently imported into the HMDB database for further analysis, resulting in the identification of 27 distinct metabolites (Table 1). Compared to the normal group, noralfentanil, artecanin, latrunculin A, 1‐tetradecanoyl‐sn‐glycero‐3‐phosphate, monopropionylcadaverine, fexofenadine, and (2E,11Z)‐wyerone acid exhibited significant upregulation. Conversely, phaseollinisoflavan, ketobemidone, chelidonine, and indicine were significantly downregulated. Following intervention with PH‐CC, these metabolites exhibited a reversal, suggesting that PH‐CC effectively ameliorates metabolic disorders in UC mice (Figure 15).

**TABLE 1:** Differential metabolites identification of mice fecal samples.

Metabolite	Mode	Normal versus Model			Model versus Treatment		
		VIP	FC	*p*	VIP	FC	*p*
Noralfentanil	pos	3.026	0.651	0.000	2.875	1.631	0.000
Monopropionylcadaverine	pos	2.880	0.617	0.000	1.709	1.214	0.000
Cabastine	pos	2.739	1.542	0.000	1.513	1.346	0.007
Chelidonine	pos	2.700	1.709	0.003	3.602	0.435	0.000
Propoxycaine	pos	2.612	0.734	0.000	2.223	1.314	0.000
Indicine	pos	2.365	1.472	0.002	3.606	0.469	0.000
Artecanin	pos	2.351	0.724	0.001	1.678	1.253	0.011
Ketobemidone	pos	2.224	1.321	0.000	1.349	0.805	0.042
Phaseollinisoflavan	pos	2.186	1.300	0.000	2.113	0.734	0.000
Tyrosyl‐Isoleucine	pos	2.151	1.318	0.000	1.434	1.336	0.042
Fexofenadine	pos	2.148	0.752	0.000	2.101	1.395	0.000
N‐Jasmonoylisoleucine	pos	2.140	1.287	0.000	1.522	1.227	0.001
Trans‐Cinnamic Acid	pos	2.027	1.334	0.002	1.339	1.236	0.013
Prochlorperazine	pos	1.916	1.230	0.000	1.546	1.245	0.001
Peimisine	pos	1.755	1.239	0.015	1.774	1.422	0.007
N‐(N‐Acetylmethionyl)dopamine	pos	1.728	1.254	0.005	1.889	1.638	0.013
Latrunculin a	pos	1.613	0.811	0.025	1.720	1.311	0.007
1‐tetradecanoyl‐sn‐glycero‐3‐phosphate	neg	2.640	0.695	0.000	1.768	1.215	0.002
AB‐MECA	neg	2.147	1.298	0.000	2.085	1.439	0.000
Valganciclovir	neg	2.040	1.245	0.000	1.934	1.324	0.000
Dihydroneopine	neg	1.982	1.264	0.000	1.455	1.321	0.042
N‐(gamma‐Glutamyl)ethanolamine	neg	1.955	1.259	0.000	1.587	1.232	0.001
Valyltyrosine	neg	1.909	1.279	0.000	1.452	1.209	0.000
(4Z,6Z)‐Octa‐4,6‐dienoylcarnitine	neg	1.790	1.213	0.000	1.909	1.333	0.000
(2S)‐2‐Amino‐3‐[(2S,3R)‐2‐amino‐3‐[(2S)‐2‐amino‐3‐(4‐hydroxyphenyl) propanoyl]oxybutanoyl]oxypropanoic acid	neg	1.691	1.216	0.001	1.559	1.233	0.001
Gabexate	neg	1.668	1.209	0.000	2.192	1.600	0.000
(2E,11Z)‐Wyerone acid	neg	1.346	0.804	0.045	1.773	1.403	0.004

**FIGURE 16:**
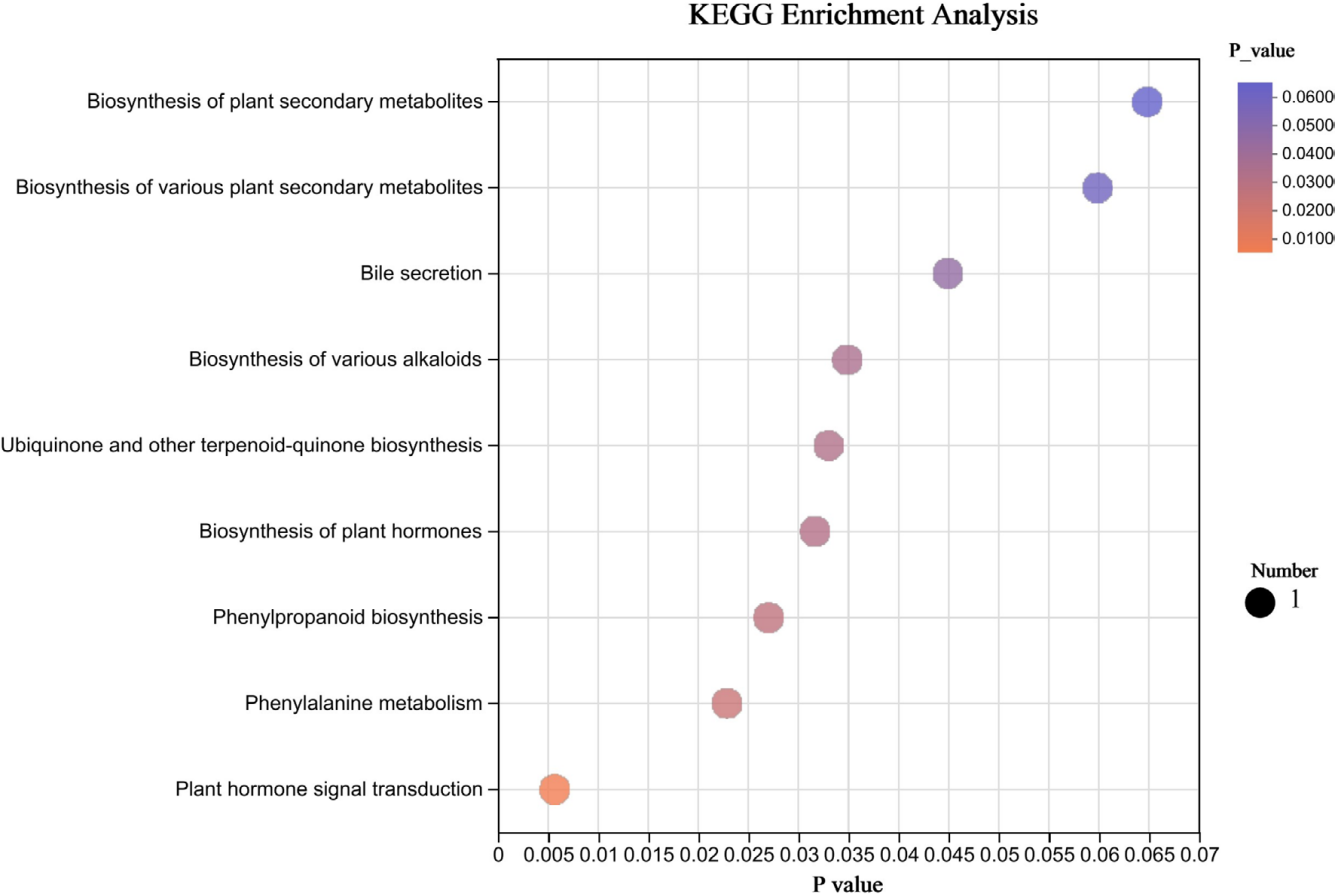
Enrichment analysis of differential metabolic pathways.

Differential metabolites in mouse fecal samples were analyzed to ascertain their involvement in metabolic pathways. A total of 27 differential metabolites were identified, associated with nine metabolic pathways (Table 2). Pathways with a significance level of *p* < 0.05 were considered potential candidates for PH‐CC intervention in UC. The metabolites were primarily linked to phenylalanine metabolism and bile secretion (Figure 16).

**TABLE 2:** Metabolic pathways involved in differential metabolites.

No.	Pathway name	Ratio_in_pop	*p*	*p*_adjust
1	Plant hormone signal transduction	12/6384	0.00563	0.05066
2	Biosynthesis of various alkaloids	75/6384	0.03484	0.05226
3	Bile secretion	97/6384	0.04490	0.05773
4	Ubiquinone and other terpenoid‐quinone biosynthesis	71/6384	0.03300	0.05940
5	Biosynthesis of plant secondary metabolites	141/6384	0.06482	0.06482
6	Biosynthesis of various plant secondary metabolites	130/6384	0.05986	0.06735
7	Biosynthesis of plant hormones	68/6384	0.03162	0.07115
8	Phenylpropanoid biosynthesis	58/6384	0.02701	0.08104
9	Phenylalanine metabolism	49/6384	0.02285	0.10280

### 3.6 Correlation Analysis Between Differential Metabolites and Intestinal Floras

**FIGURE 17:**
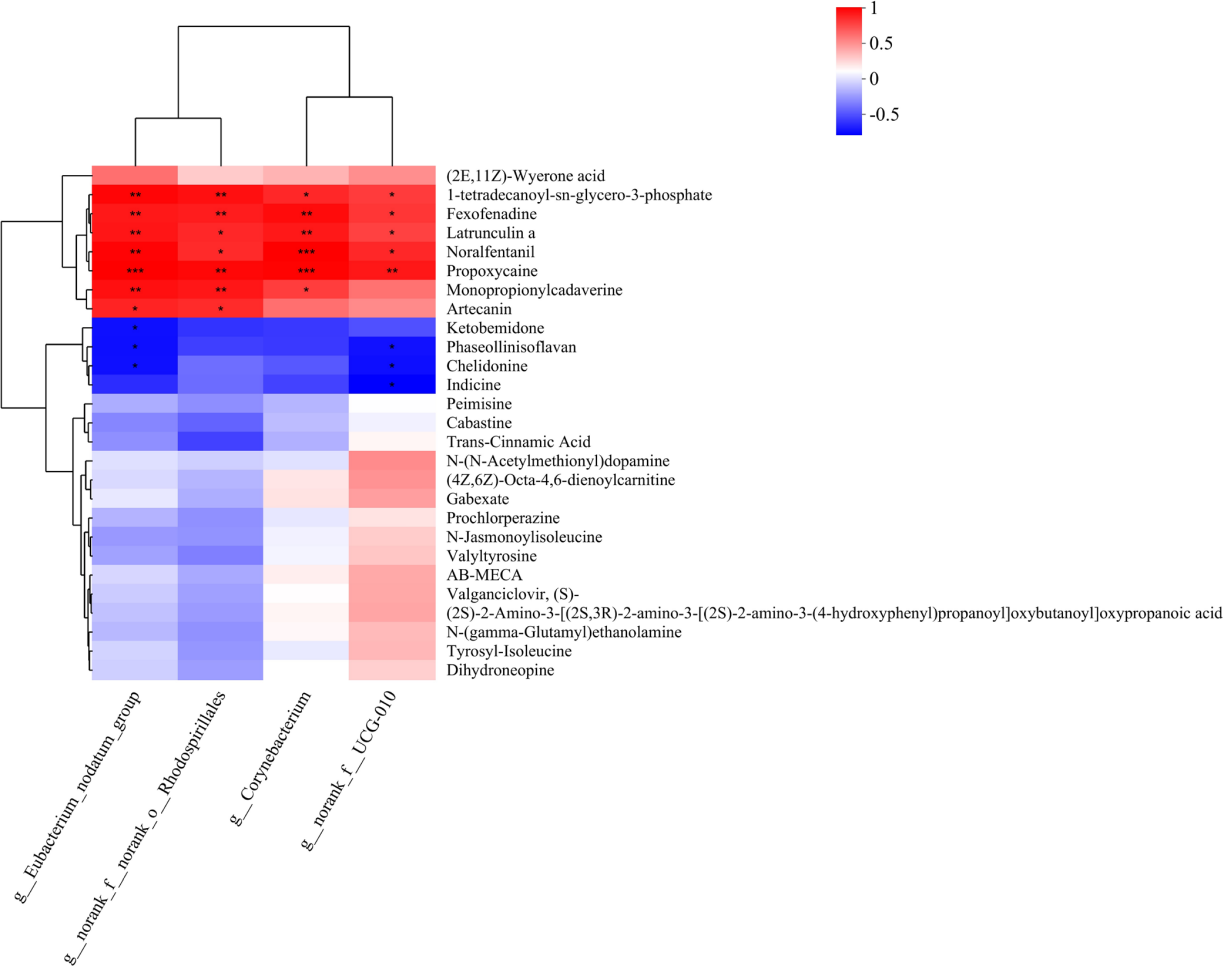
Correlation analysis between intestinal floras and differential metabolites.

To further investigate the relationship between differential metabolites and intestinal flora, Pearson correlation analysis was conducted to assess the correlation between the identified differential floras and the 27 specific metabolites at the genus level. The genera *Norank_f_norank_o_Rhodospirillales, corynebacterium, norank_f_UCG‐010*, and *eubacterium_nodatum_group* exhibited positive correlations with metabolites like noralfentanil, latrunculin A, and 1‐tetradecanoyl‐sn‐glycero‐3‐phosphate. Conversely, these same floras demonstrated negative correlations with metabolites including phaseollinisoflavan, chelidonine, ketobemidone, and indicine (Figure 17).

## 4 Discussion

In this study, a 3% DSS solution was utilized to induce UC in mice. Histopathological analysis revealed that mice exposed to DSS exhibited significant damage to the crypt structure, as well as disruption and detachment of the colonic mucosal epithelium, glandular loss, and extensive infiltration of inflammatory cells. Treatment with PH‐CC was observed to mitigate colonic tissue damage in these mice. These findings suggest that PH‐CC may effectively reduce inflammatory responses and alleviate colonic mucosal injury in UC models. Oxygen free radicals play a crucial role in the pathogenesis of UC, as they can cause lipid peroxidation of cell membranes, damaging cellular structure and function, which leads to intestinal mucosal damage and inflammatory reactions (Sahoo et al. 2023). The results of antioxidant activity demonstrated that PH‐CC exhibited strong reducing capability and effectively scavenged DPPH free radicals and hydroxyl radicals, showing a significant dose–response relationship. These results indicate that PH‐CC possesses a robust antioxidant effect in vitro. Furthermore, diversity analysis of bacterial communities between groups revealed a high degree of separation, indicating a strong association between DSS‐induced UC and alterations in intestinal flora in mice. Importantly, PH‐CC intervention was found to restore the composition of the flora community in UC mice.

To investigate the potential therapeutic impact of PH‐CC on UC and its possible relationship with the regulation of intestinal flora, we conducted an analysis of the intestinal flora composition in mice. Our findings revealed that mice with DSS‐induced UC exhibited a notable increase in the relative abundance of *norank_o_Rhodospirillales, ruminococcaceae_UCG‐010*, and corynebacteriaceae at the family level. Following intervention with PH‐CC, there was a decrease in the abundance of these specific taxa. While previous studies have not directly linked *ruminococcaceae_UCG‐010* to inflammatory bowel disease, research has indicated that alterations in its abundance can lead to increased deoxycholic acid biosynthesis, an unbound secondary bile acid associated with inflammatory responses (Biagioli et al. 2021; Qu et al. 2022). Furthermore, DSS‐induced UC mice displayed a significant increase in the relative abundance of *norank_f_norank_o_Rhodospirillales, norank_f_UCG‐010, corynebacterium*, and *eubacterium_nodatum_group* at the genus level, which were subsequently downregulated by the PH‐CC intervention. Our results suggest that PH‐CC may modulate intestinal flora, including the *eubacterium_nodatum_group* and *corynebacterium*, to ameliorate UC symptoms. *Corynebacterium* is known to significantly impact human intestinal microbiota composition and can induce local inflammation, tissue damage, and systemic toxicity through toxin secretion. Clinical studies have emphasized the potential of corynebacterium to cause various diseases, particularly in immunocompromised individuals. Additionally, the eubacterium_nodatum_group and its metabolite short‐chain fatty acids play a role in regulating the release of inflammatory factors (Li, Li et al. 2023; Li, Zhu et al. 2023). Eubacteria_nodatum_group is commonly found in anaerobic infections, which have been identified as potential triggers for the development of inflammatory bowel disease (Martínez de Victoria Carazo et al. 2023; Rashed et al. 2022). Overall, our study findings align with existing literature.

Metabolomics analysis identified a total of 27 differential metabolites, with PH‐CC intervention significantly modulating key metabolites such as fexofenadine, chelidonine, and indicine. Chelidonine, classified as a benzophenanthridine alkaloid, has been demonstrated to potentially inhibit IL‐1β‐induced inflammatory responses both in vitro and in vivo by modulating the TLR4/NF‐κB pathway (Li, Li et al. 2023; Li, Zhu, Shao et al. 2023). Fexofenadine, known as a novel inhibitor of TNF‐α signaling, exhibits therapeutic effects on inflammatory bowel disease (Zhao et al. 2021). These metabolites are involved in metabolic pathways, including bile acid metabolism and phenylalanine metabolism. Bile acids, which are essential components of bile, play a significant role in the development of inflammatory bowel disease by interacting with bile acid receptors and regulating intestinal flora (Calzadilla et al. 2022; Jia et al. 2024; Yang, Gu, et al. 2021). Perturbations in bile acid metabolism are observed in patients with inflammatory bowel disease, where the overexpression of bile acids and the metabolic enzyme CYP8B1 can exacerbate intestinal barrier damage and impair repair functions (Chen et al. 2022). The Chinese herb oregano has been found to attenuate DSS‐induced inflammatory responses, enhance intestinal barrier repair, promote bile acid absorption in UC mice, and regulate intestinal bile acid metabolism to maintain intestinal mucosal immune homeostasis (Yu et al. 2023). Phenylalanine, an essential amino acid, influences immune responses and intestinal barrier function through its metabolism. Studies suggest that phenylalanine and its metabolites such as phenylacetylglutamine are involved in the regulation of inflammatory bowel disease by activating nuclear factor‐E2‐related signaling pathways or platelets (Yao et al. 2025; Jialing et al. 2020; Pang et al. 2025; Zhou et al. 2024).

The analysis of the relationship between intestinal flora and pathological changes, inflammatory factors, and metabolites reveals significant findings. The results from gut microbiota assessments and histopathological examinations indicate a notable increase in the abundance of *norank_f_Rodospirillales, norank_f_UCG‐010, Corynebacterium*, and *Eubacterium nodatum groups* within the model group, which coincides with severe damage to the colonic mucosa and extensive infiltration of inflammatory cells (Krueger et al. 2025). Following PH‐CC intervention, there was a marked decrease in the abundance of these microbiota, accompanied by a significant improvement in colonic mucosal damage. These findings suggest a strong association between these four specific microbiota and colonic mucosal injury in UC mice. Furthermore, PH‐CC may regulate intestinal flora dysbiosis and mitigate UC‐related colonic mucosal damage by downregulating the abundance of these four differential microbiota.

The abnormal activation of the NLRP3/Caspase‐1 pathway is a critical factor in the development of UC (Qi et al. 2024; Zhang et al. 2022; Wang et al. 2022). Our previous research has demonstrated that PH‐CC can mitigate UC by inhibiting the abnormal activation of this pathway and the subsequent release of downstream inflammatory factors. To investigate the potential impact of intestinal flora on the activation of the NLRP3/Caspase‐1 pathway and the release of inflammatory factors, a correlation analysis was conducted. The results revealed a positive association between four differential intestinal flora (*norank_f_UCG‐010, eubacterium_nodatum_group, norank_f_norank_o_Rhodospirillales and corynebacterium*) and key components of the pathway (NLRP3, Gasdermin D, and Caspase‐1), as well as downstream inflammatory factors (IL‐18, IL‐1β, and MPO). It is hypothesized that disturbances in intestinal flora may influence the activation of the NLRP3/Caspase‐1 pathway, leading to an increased release of inflammatory factors and subsequent UC‐related colon mucosal inflammation. Additionally, PH‐CC is believed to exert its therapeutic effects by modulating gut microbiota disorders, suppressing abnormal pathway activation, and reducing the release of inflammatory factors. Studies have revealed a notable association between streptococcus, Escherichia‐Shigella, and the expression levels of IL‐6 and TNF‐α in DSS‐induced UC mice, indicating a link between inflammatory factor levels and intestinal flora imbalance (Feng et al. 2023). The findings of this study align with existing literature reports. Furthermore, the correlation analysis between intestinal flora and differential metabolites highlighted a strong relationship between *norank_f_norank_o_Rhodospirillales, eubacterium_nodatum_group*, *norank_f_UCG‐010*, and *corynebacterium* with differential metabolites, suggesting a potential role of intestinal flora in regulating host metabolic processes. Based on these findings, it is hypothesized that *norank_f_norank_o_Rhodospirillales, corynebacterium, eubacterium_nodatum_group*, and *norank_f_UCG‐010* may be key intestinal microorganisms influencing UC.

## 5 Conclusion

In summary, PH‐CC has the potential to alleviate UC by correcting the imbalance of intestinal flora and downregulating the relative abundance of *norank_f_norank_o_Rhodospirillales*, *corynebacterium, eubacterium_nodatum_group*, and *norank_f_UCG‐010*. This action mitigates inflammatory damage to the colonic mucosa. These findings provide an experimental foundation for understanding how PH‐CC can ameliorate UC through the regulation of intestinal microorganisms.

## Author Contributions

**Feifei Zhu:** data curation (equal), writing – original draft (equal). **Chenhui Ren:** formal analysis (equal), software (equal), writing – original draft (equal). **Keli Zhou:** data curation (equal), formal analysis (equal), software (equal), visualization (equal). **Xueqing Huang:** data curation (equal), formal analysis (equal), software (equal). **Mengyu Mei:** software (equal), visualization (equal). **Haiyan Niu:** funding acquisition (equal), resources (equal), supervision (equal), writing – review and editing (equal). **Shouzhong Ren:** conceptualization (equal), funding acquisition (equal), methodology (equal), writing – review and editing (equal).

## Conflicts of Interest

The authors declare no conflicts of interest.

## Data Availability

The data presented in this study are available in the article.
